# Resolution-Enhancing Structure for the Electric Field Microsensor Chip

**DOI:** 10.3390/mi12080936

**Published:** 2021-08-07

**Authors:** Xiaolong Wen, Pengfei Yang, Zhouwei Zhang, Zhaozhi Chu, Chunrong Peng, Yutao Liu, Shuang Wu, Bo Zhang, Fengjie Zheng

**Affiliations:** 1 Beijing Engineering Research Center of Detection and Application for Weak Magnetic Field, Department of Physics, University of Science and Technology Beijing, Beijing 100083, China; xiaolongwen@ustb.edu.cn (X.W.); bzhang@ustb.edu.cn (B.Z.); 2 School of Applied Science, Beijing Information Science and Technology University, Beijing 100192, China; 3 State Key Laboratory of Transducer Technology, Aerospace Information Research Institute, Chinese Academy of Sciences, Beijing 100190, China; zhangzhouwei15@mails.ucas.ac.cn (Z.Z.); crpeng@mail.ie.ac.cn (C.P.); fjiezheng@163.com (F.Z.); 4 Institute of Microelectronics of Chinese Academy of Sciences, Beijing 100029, China; chuzhaozhi@ime.ac.cn; 5 Beijing Tflying Transducer Technology Co., Ltd., Beijing 100083, China; ytaoliu@tflying.cn (Y.L.); wushuang@tflying.cn (S.W.)

**Keywords:** electric field, electric field microsensor, package, vibrating capacitor, electrostatic charge

## Abstract

Electrostatic voltage is a vital parameter in industrial production lines, for reducing electrostatic discharge harms and improving yields. Due to such drawbacks as package shielding and low resolution, previously reported electric field microsensors are still not applicable for industrial static monitoring uses. In this paper, we introduce a newly designed microsensor package structure, which enhances the field strength inside the package cavity remarkably. This magnification effect was studied and optimized by both theoretical calculation and ANSYS simulation. By means of the digital synthesizer and digital coherent demodulation method, the compact signal processing circuit for the packaged microsensor was also developed. The meter prototype was calibrated above a charged metal plate, and the electric field resolution was 5 V/m, while the measuring error was less than 3 V, from −1 kV to 1 kV in a 2 cm distance. The meter was also installed into a production line and showed good consistency with, and better resolution than, a traditional vibratory capacitance sensor.

## 1. Introduction

Electrostatic voltage is a key parameter in industrial production lines, which quantitatively indicates the static charge in products. When it is exceeded, electrostatic discharge (ESD) most likely happens to cause direct breakdown or invisible inner damage [1]. Although ESD protection technologies have been taken into consideration in the circuit designs [2,3,4], in the preliminary stages of manufacturing, the fundamental elements are still under risks of ESD failure. For instance, in the organic light-emitting diode (OLED) screen factories, static charge might arise to 5000 volts on glasses, after such manufacturing processes as surface cleaning, physical vapor deposition, and photoetching [5,6,7]. The batch production of integrated circuits and surface mount technology (SMT) processes also face similar problems [8]. Antistatic material-based facilities, corona ionizers, and soft X-ray ionizers, are the most used methods for preventing static charge accumulation [9,10,11]. Nevertheless, the real-time effectiveness of these methods is unclear, due to a lack of online detection means. The breakdown of antistatic facilities can be only found in the offline periodic examination or calibration. A preferable method for evaluating the static charge is the long-term electrostatic voltage measurement, which not only shows the quantity of static charge directly, but also gives clear evidence about the antistatic facility failure [12,13]. With the increasing density of integrated circuits in recent years, the electrostatic protection is becoming more rigorous, meanwhile more attention is being paid to electrostatic voltage measurement in the manufacturing processes.

The electric field mills and vibrating capacitors are the most popular contactless methods that detect the surface charge. Both of these utilize an A.C. carrier type system, to modulate the capacitance pickup in the measured electric field. In an electric field mill, there is a grounded rotor-driven shielding electrode that chops the electric field; therefore, the induced charge on the sensitive electrode is modulated into an alternative current for demodulation [14,15,16,17]. Because of the complex mechanical structure, its drawbacks include its bulky and limited motor life. The frequency response is also limited, due to the low angular velocity of the motor. The vibrating capacitor type is made of a metal plate and a piezoelectric actuator. While an alternating voltage is applied to the actuator, the metal plate periodically vibrates, resulting in an alternative induced current for demodulation [18,19,20,21]. Because of the higher modulation frequency than the field mill, the vibrating capacitor shows a better response time performance. Moreover, benefiting from the vibration mechanism, it avoids abrasion in comparison with the motor-driven field mill, and therefore has a longer working life. Thus, the vibrating capacitor type is prevailing in those portable and long-time static meter products. Nevertheless, this type still needs precision assembly processes, which limits the batch applications. The piezoelectric coefficient of the piezoceramics actuator changes, along with temperature and time, and the resonance of the piezoceramics actuator is easily affected by the dusty air; therefore, most vibrating capacitors are not suggested to be used in those harsh environments.

Based on micro-electro-mechanical systems (MEMS) technology, the resonant electric field microsensor (also known as the micro field mill) employs a similar working principle, but has a lot of advantages, such as small, light, low-power consumption, wear-free, batch manufacturing, etc. The study on the resonant electric field microsensor began in the 1990s, to our knowledge. Since then, the electric field resolution is being improved, from 630 V/m to 20.4 V/m, by various innovative micro sense structures [22,23,24,25,26,27,28,29,30,31,32,33,34,35,36,37], under the parallel plate calibration. The electric field microchips were mostly exposed to the external environment for test, historically. Nevertheless, the resolution of the electric field is still insufficient for those low static voltage sensing applications, due to their small sensing area and weak induced current. Furthermore, the resonant microchip might be damaged without protection, and the signal processing system is too bulky to be installed in those narrow spaces [38]. The package of the electric field microsensor is also important, but is not fully considered yet. Several previously reported packaging prototypes were prone to shield the outer electric field, by the package cap and sidewalls of the package cavities, therefore causing an attenuation of field resolution [39,40]. In on our previous work [41], we found the effectiveness of a non-flat package structure and reported some measured results.

Different from previous reports, in this paper we study the impact of the microsensor package structure to the local electric field distribution, and enhance the field strength inside the package cavity, to gain an ultra-high resolution by an innovative taper shape package cap. Also, by means of digital driving and a signal demodulating circuit, a compact static meter prototype is developed, calibrated, and then applied in an OLED manufacturing line [42].

## 2. Materials and Methods

This paper proposes a new package structure for the electric field microsensor, and the resolution is enhanced remarkably. We also developed a compact circuit for the microchip, and ran a series of tests on the meter prototype.

### 2.1. Principle of the Electric Field Microsensor

**Figure 1:**
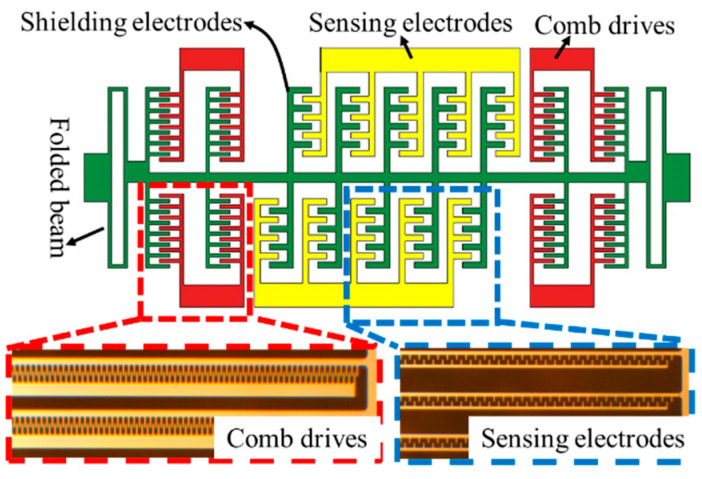
Sketch and pictures of the electric field microchip.

Figure 1 shows the sketch and the photo of the electric field sensor (EFS) based on MEMS technology, consisting of two sense electrodes, an earthed resonant shield electrode, a driven electrode, and a folded beam. From the vibration of shield electrode, time-varying electrical charge signal is induced on the sense electrodes whereas the signal amplitude is proportional to the measured field. Two groups of differential sensing electrodes are designed to eliminate the cross-interference from the surrounding circuits. As reported by our group [25,29,30], the EFS is fabricated from a silicon-on-insulator (SOI) wafer, where all the components shown in Figure 1 distribute in the same plane.

### 2.2. Package Structure Design and Simulation

Previously reported sealed packages mainly contained a flat package cap on an insulative support. The flat cap is either metallic or insulative, either of which formed a float-potential body for the electric field to pass through. Although the vulnerable microchip was well protected, the electric field penetrating the package cap is attenuated too, therefore causing the resolution to be worse.

The relationship between the electric field inside and outside the package cavity is given by Equation (1), as follows:

(1) $$E_{in}=c\cdot E_{out}$$

where the $E_{in}$ and $E_{out}$ are the electric field in and out of the package cavity, respectively, and c is the amplification coefficient, which is usually less than 1 in existent reports.

**Figure 2:**
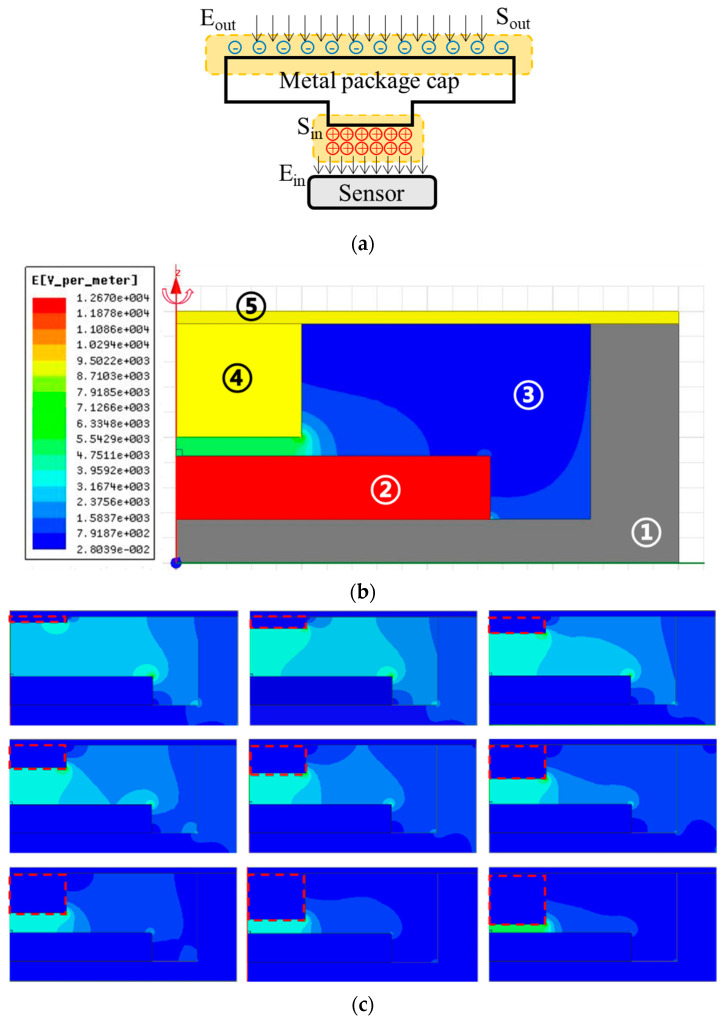

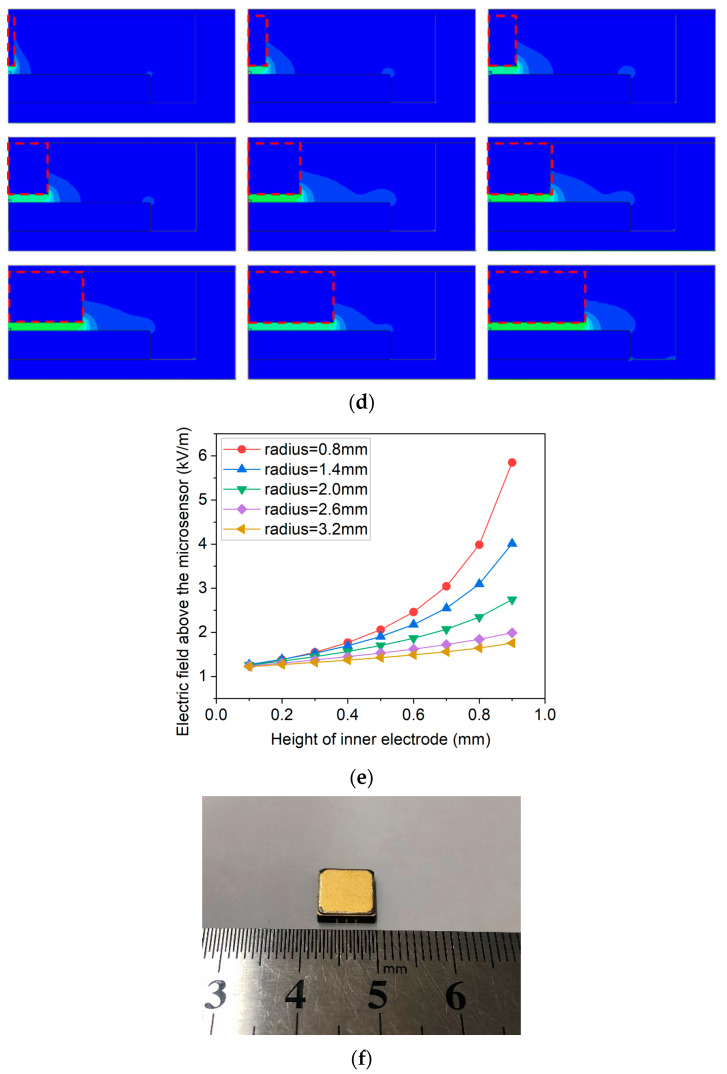
Package design of the electric field microchip. (**a**) Mathematical analysis model of the microsensor package. The model only includes the package cap and the microchip while the package shell and the gold wires are neglected to simplify the calculation. (**b**) Electric field nephogram simulation results on the enhancement effect of package. (**c**) Field nephogram comparison when the height of the inner electrode changed from 0.1 mm to 0.9 mm. (**d**) Field nephogram comparison when the width of the inner electrode changed from 0.1 mm to 1.7 mm. (**e**) Simulated results of the relationship between the dimensions and the electric field. We changed the radius and the height of the inner electrode, and meanwhile a small air dome was created to read the electric field above the microsensor. (**f**) Picture of the sealed cavity using ceram material as the package shell, and covar as the package cap. Total size of the packaged chip is 8 mm × 8 mm × 2 mm.

Here, we take the metal package cap for calculation, as shown in Figure 2a. The fringe effect of charge distribution and the support for the metal package cap are neglected in this model. Since the cap is float potential, the induced charge on the top and bottom of the metal package cap in Figure 2 are equal in quantity, but opposite in sign. Based on the electric field Gauss theorem, the integrals around the two surfaces are written as follows:

(2) $$\oint_{S_{out}}E_{out}ds=\frac{Q_{out}}{\varepsilon_{0}}$$

(3) $$\oint_{S_{in}}E_{in}ds=\frac{Q_{in}}{\varepsilon_{0}}$$

where the $Q_{out}$ and $S_{out}$ represent the charge and area on the top, respectively. $Q_{in}$ and $S_{in}$ are defined in the same way on the bottom surface. Since $Q_{out}=-Q_{in}$, and the electric field inside the metal package cap is zero, from (2) and (3) we can get the following:

(4) $$E_{in}=\frac{S_{out}}{S_{in}}\cdot E_{out}$$

Equation (4) shows that the amplification coefficient c is decided by the area ratio of the two surfaces. For the previous flat cap structure, c=1 in this model. However, if the support for the cap is further considered and calculated, the amplification coefficient c<1.

In order to gain a high-sensitive packaged microsensor, a new package structure is proposed by using Equation (4). Different from the simple flat structure, the new concept has designed a taper shape package cap, in which the out surface is large enough to collect the induced charge, and the inside surface is small to concentrate the opposite induced charge. Therefore, the electric field can be enhanced inside the package cavity.

In order to examine the theoretical analysis and find the influence of the package on the local electric field distribution around the microsensor, we made an electrostatic simulation for the proposed package structure. Figure 2b shows the finite element simulation model and electric field nephogram in ANSYS Maxwell 16.0 for studying the effectiveness of the conception. The simulated model is constituted by the package shell, MEMS chip (simplified into a solid structure from Figure 1) fixed inside the shell, air inside the shell, inner electrode, which connects to the package cap, numbering from 1 to 5, respectively. The model was designed axially symmetric, and the only variable was the width or the height of the inner electrode, as the enhancement was mainly decided by the area of the package cap. The detailed size and material of the model is shown in Table 1.

Besides the package model, a five-times longer and wider air dome was also modeled, and the excitation voltage on the air dome was 22 volts in order to generate the electric field of 1 kV/m. The solution type of the simulation was electrostatic type. As the model was designed axially symmetric, the geometry mode was cylindrical about Z-axis. We used the triangle type of elements, whose maximum length was set as 0.01 mm to guarantee the accuracy of solution. The convergence criteria were as follows: percent refine: 30; minimum number of passes: 2; minimum coverage passes: 1; maximum number of passes: 10; percent error: 1.

It can be observed from the simulated nephogram that the electric field around the proposed package structure is distorted, and the electric field strength between the inner electrode and the MEMS chip was enhanced significantly by one order of magnitude, while the other places were attenuated by the package structure. To study the effect of such structure, a series of simulations were made with different radiuses and heights of the inner electrode, and the corresponding electric field inside the package gap is shown in Figure 2c–e. The series of curves implies that we can enhance the local electric field and then improve the resolution by minishing the diameter of the inner electrode. The relationship between the radius of the inner electrode and the electric field agrees well with the theoretical analysis in Equation (4). The simulated curves suggest that the resolution can also be enhanced by reducing the gap distance between the inner electrode and the MEMS chip, and it is also reasonable because the influence from the sidewall of the package shell is relatively smaller when the gap is narrower.

As shown in Figure 2f, a highly sensitive air tight package was fabricated. It was constituted of a covar cap, a ceram shell and an inner electrode. The gas tightness was examined by the helium mass spectrometer leak detector, and the gas leakage rate was better than $1.1\times 10^{-9}\;\mathrm{Pa}\cdot m^{3}/s$. The airtight package was fabricated by the following steps. The inner electrode was welded to the inner surface of the cap with hard-solder at the first step. Considering the area of the microchip sensing part, the diameter of the inner electrode was 2 mm. Also, for guaranteeing the safety, the height of the electrode was 0.78 mm and the gap distance was 0.15 mm. After the MEMS chip was bonded to the ceram shell with conductive silver adhesive and wired to the bonding pad, the cap was connected to the package shell by the parallel seam welding process. 

To further enhance the measured electric field, an outer electrode, which connects the package cap with a metal wire to collect more induced charge, is also proposed. The outer electrode is exposed to the measured electric field outside, and can be supported by the shell of the whole meter with insulative material. Therefore, its area is to the magnitude of the voltage meter. Benefiting from the optimization on the shape of package and the outer electrode connected to the package cap, local electric field near the sense chip inside the sealed space is remarkably strengthened rather than attenuated as before.

Although the induced charges on the package cap are calculated, they are prone to leak to a lower potential through the isolating dielectric. Besides, some of the space charges will be directly trapped by defects or interfaces in the dielectric, and there is also a leakage current to the lower potential. Further, the metal cover plate might carry extra external charges in an ionic environment, while the extra charges also decay with time.

The leakage current can be expressed by the full current formula as shown in Equation (5).

(5) $$J=J_{c}+J_{d}$$

where $J_{c}$ is the conduction current density and $J_{d}$ is the displacement current density. Assuming that the packaging dielectric material is polarized, isotropic, and stable in resistivity, it can be obtained according to the microscopic form of Ohm’s law and the displacement current formula in Equations (6) and (7).

(6) $$J_{c}=E/\rho$$

(7) $$J_{d}=\varepsilon \frac{\partial E}{\partial t}$$

The above E is the electric field strength, ρ is the resistivity, and ε is the dielectric constant of the dielectric volume. Since the system is at an open-loop state when the surface charge of the medium starts to dissipate, and the full current is zero at this time, the following result can be obtained, as shown in Equation (8).

(8) $$\frac{E}{\rho }+\varepsilon \frac{\partial E}{\partial t}=0$$

Thus, the decay of the electric field strength with time can be solved by the following:

(9) $$E\left(t\right)=E_{0}*e^{-t/\rho \varepsilon }$$

where $E_{0}$ represents the initial electric field strength. The material used in the experiment is ceram, and its reference values of bulk resistivity and relative dielectric constant are $\rho =10^{14}\;\Omega m$, $\varepsilon_{r}=9$, respectively.

### 2.3. Signal Processing Circuit and Demodulation Algorithm Design

**Figure 3:**
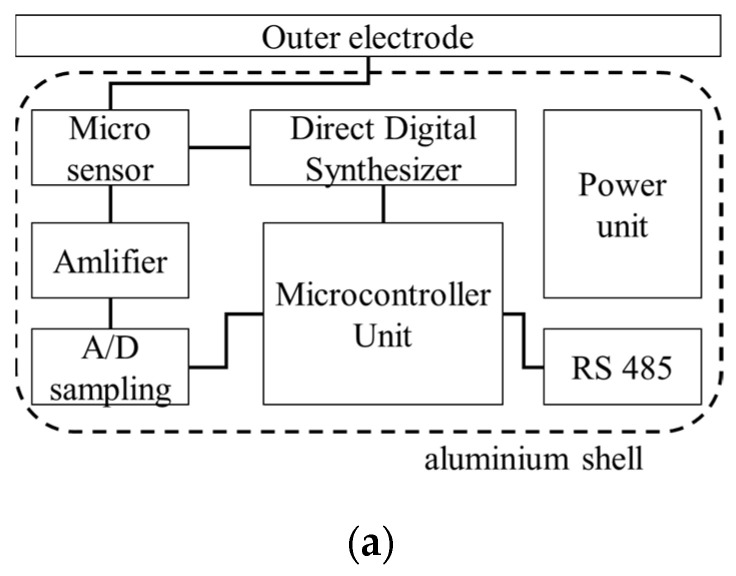

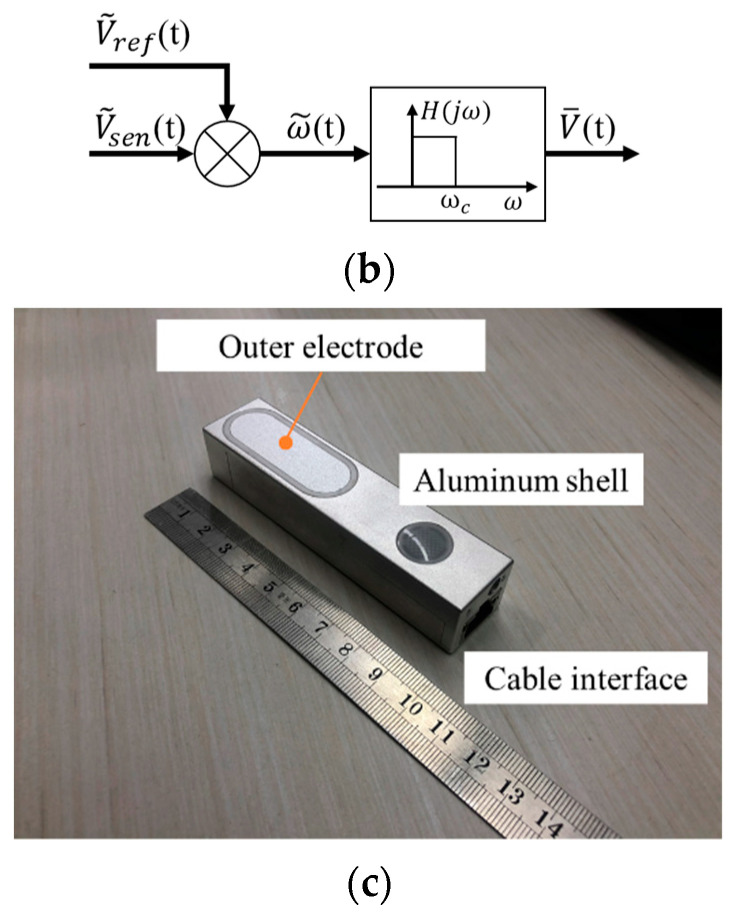
Signal processing circuit of the microsensor. (**a**) Circuit diagram. (**b**) Phase-sensitive demodulation algorithm. (**c**) Photo of the electrostatic meter prototype.

The signal processing circuit includes the amplifier, A/D sampling chip, etc., shown in Figure 3a. The resonant driving electrostatic force is generated by the direct digital synthesizer (DDS), which is controlled by the microcontroller unit (MCU). Regarding the detecting part, the weak output alternative current of the microsensor is first magnified by a transimpedance amplifier, and then magnified by an instrument amplifier. High-speed and high-accuracy A/D sampling chip AD7660 is used to digitize the magnified microsensor output ${\tilde{V}}_{sen}$ and the driving voltage ${\tilde{V}}_{ref}$, as expressed by Equations (10) and (11), respectively.

(10) $${\tilde{V}}_{sen}=k_{E}Ecos\left(\omega t+\theta \right)$$

(11) $${\tilde{V}}_{ref}=V_{1}\cos\left(\omega t\right)$$

where $k_{E}$ and E represent the sensitivity and measured electric field, $V_{1}$ is the amplitude of the alternative driving voltage, ω is the resonant frequency of the microsensor, and θ is the phase difference of these two signals. The unique feature of the signal processing circuit is that the amplitude of the alternative signal is demodulated by the digital phase-sensitive method, which mainly contains a multiplication and a low-pass filter algorithm, as shown by Figure 3b. The product of ${\tilde{V}}_{sen}$ and ${\tilde{V}}_{ref}$ is given by Equation (12), which consists of a direct and an alternative voltage part with a double frequency. After the low-pass filter, the alternative voltage is removed while the final output is shown in Equation (13). The final output voltage is in proportion to the measured electric field.

(12) $$\tilde{\omega }\left(t\right)=\frac{1}{2}k_{E}EV_{1}\cos\theta +\frac{1}{2}k_{E}EV_{1}\left(\cos2\omega t\cos\theta -\sin2\omega t\sin\theta \right)$$

(13) $$\bar{V}=\frac{1}{2}k_{E}EV_{1}\cos\theta$$

The advantages of this digital demodulation algorithm include anti-interference from the environment and fast processing given the high-speed MCU. The meter’s measuring results are transmitted in accordance with RS485 protocol. The photo of the proposed electrostatic meter prototype is shown in Figure 3c, and the total power of the meter is 600 mW, the size of the sensor prototype is 99 mm × 23 mm × 19 mm.

## 3. Results

### 3.1. Laboratory Calibration and Accuracy Test Results

**Figure 4:**
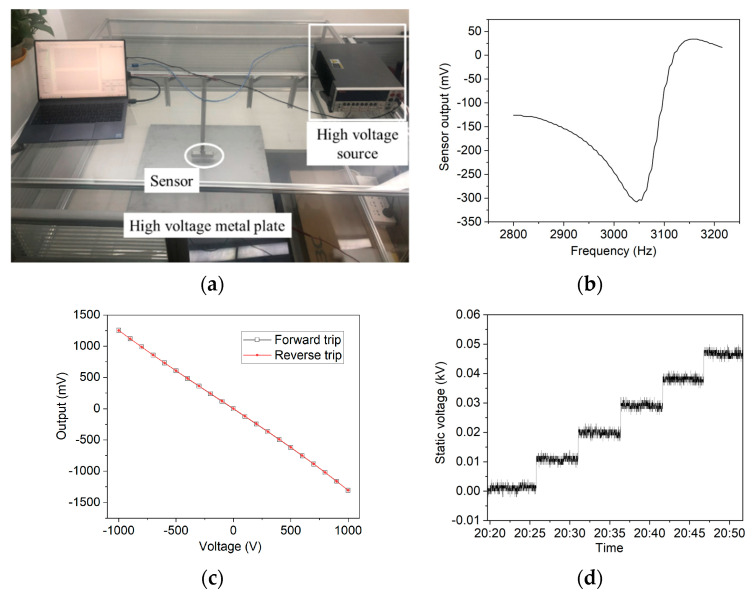
The electrostatic voltage calibration system and results of the sensor. (**a**) Picture of the calibration system, including the high-voltage source, the metal plate of 0.6 m × 0.6 m, the sensor’s metal support, and the computer. (**b**) The amplitude-frequency characteristic of the resonant microchip. The frequency ranged from 2800 Hz to 3200 Hz, and the resonant frequency shown by the curve was 3045 Hz. (**c**) Calibration results of the meter prototype above the charged metal plate. The voltage generated by the Keithley 2410 changed from −1 kV to 1 kV and then changed backwards to examine the hysteresis of the prototype. (**d**) Long-term accuracy measurement results after calibration. Six voltages, equally ranging from 0 V to 50 V, were applied on the metal plate, and in each voltage five minutes’ results were recorded. The distance between the meter and the plate was 2 cm.

The calibration system consisted of a high voltage power supply and a metal plate, as shown in Figure 4a. The meter was fixed perpendicularly to the plate, with an electrically grounded aluminum support, while the distance between them was adjustable. The high voltage power was Keithley 2410, and the maximum output voltage range was from −1.1 kV to +1.1 kV.

The resonant frequency of the sensor was first tested by the digital frequency sweep method. The MCU controlled the DDS, to generate a continuously changing frequency, and meanwhile demodulated the output of the sensor, to find out the maximum or minimum value. Figure 4b shows the amplitude-frequency characteristic of the developed static meter, and the maximum absolute amplitude was at 3045 Hz. Therefore, the resonant microsensor’s frequency was set to this constant value. The resonant frequencies of different microsensors vary slightly, ranging from 2800 Hz to 3200 Hz roughly, because the dimensions of each resonator differ from each other, causing a different mass, and this might also be because the internal stress differs, causing a different stiffness. Under the resonant frequency, the shielding electrode in the micro structure has the largest amplitude, and the output of the sensor changes the most from the original value.

In the meter calibration, the gap distance between the meter and the plate was set as 1 cm. By applying step voltages, from −1 kV to 1 kV, and then backward, the output of the meter was recorded synchronously. The slope of the fitting straight line was calculated as the sensitivity. Figure 4c shows the calibration results when applying voltages ranging from −1 kV to 1 kV. The output of the meter before the calibration was in mV, which represented the amplitude of the alternative voltage sampling from the amplifier. It is shown that the forward trip results agree well with the reverse trip, and the R-square value of the linear fit was 99.96%, indicating that this meter had a good performance at hysteresis and accuracy. The results of the calibration proved two aspects. First, the package of the microsensor was effective at electric field sensing, without fast charge leakage. In contrast, if there was an obvious leakage current from the package cap to the electrical ground, the output of the sensor might attenuate fast to zero. Second, the signal processing circuit was effective at demodulating the output of the microsensor, otherwise the linearity of the fitting line might be worse.

In the accuracy test, the gap distance was set to 2 cm. Before the test, the cite geometrical coefficient needs to be calibrated, because the distance was changed. This could be accomplished easily by applying a known voltage to the plate, and calculating the multiple between the input and the output direct voltage. Figure 4d shows the long-term accuracy test curve. The gap distance was set to 2 cm, and the cite geometrical coefficient was then calibrated before the real test. When the applied voltage changed from 0 V to 50 V, at an interval of 10 V, for five minutes each, the measured results showed a good accuracy with an absolute error less than 3 V. The error could further be improved if subsequent data smoothing processing was added. The resolution of the sensor prototype was also measured by the parallel-plate electric field source, in which the electric field is calculated by dividing the applied voltage by the parallel-plate distance. Similarly as with the measured curve in Figure 4d, we found that the resolution of the electric field microsensor was 5 V/m. Compared with the unpacked microsensor’s result, reported before in [30], under the same calibration system, the measured results of the electric field resolution and the electrostatic voltage error show that the proposed package was effective at improving the resolution performance.

### 3.2. The Charge Leakage in Package

To examine the influence of charge leakage of the MEMS electric field sensor in measurements, we used, in the structure, a group of corona wires to generate an ion current. By applying a positive high voltage to the corona wire in the air, to carry out corona discharge, ions such as ${\mathrm{NO}}^{+},\;{\mathrm{NO}}_{2}{}^{+},\;H^{+}$, etc., were thus generated [11]. After these ions passed through the ion flow control plate and the lower metal plate, a uniform ion flow field was formed between the upper and the lower metal plates. A Wilson plate was utilized to measure the current density that was generated by the ion current at the upper plate, and the electric field sensor was placed near the upper plate. 

In order to obtain an obvious ion current, the voltage that was applied at the corona wire was 25.5 kV, and the electric field intensity that was applied between the upper and the lower plates was E=10 kV/m. At the beginning of the experiment, the Wilson plate measured the ion current that was generated by corona, while the electric measured by electric field sensor increased rapidly, and the increasing speed dropped slowly. At about 40 min, the speed of the electric field increase dropped rapidly, and stabilized after 2 h. When the measured value of the electric field was stable, all the voltages were turned off, to allow the accumulated charge to dissipate freely.

**Figure 5:**
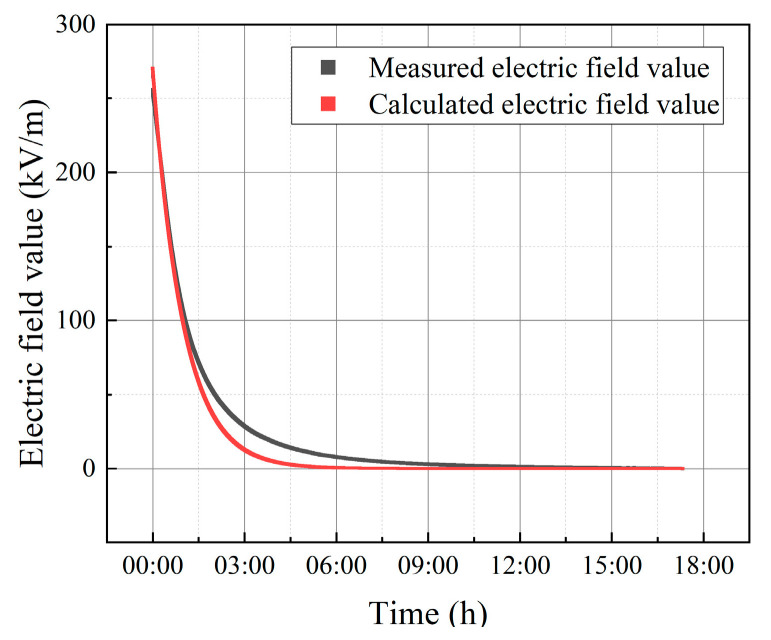
Comparison of the measured and calculated electric field values in charge decay.

In the process of charge attenuation, the initial value of the electric field was $E_{t=0}=269.959\;\mathrm{kV}/m$. The comparison between the electric field value that was measured by the sensor and the electric field value that was calculated using the above model formula, is shown in the following Figure 5.

As can be observed from the figure, the electric field change curve that was calculated by using the bulk charge attenuation, was generally consistent with the measured data, while the attenuation time constant of the measured data was larger than that calculated by the model, which may be due to the change in the bulk resistivity and dielectric constant of the material during the actual charge attenuation process. In addition, ion neutralization in the air will also affect the attenuation curve.

### 3.3. Real Test Results in the OLED Manufacturing Line

**Figure 6:**
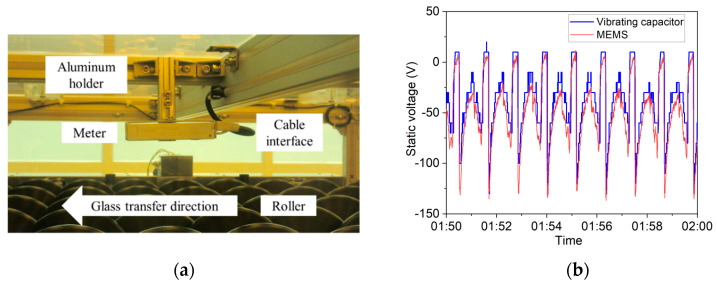
Real test installation and results. (**a**) Picture of the probe installed in the OLED product line. The glass moved beneath the meter by the roller, and the measure distance was 5 cm. The aluminum holder and the meter were both electrically grounded together. One single net cable was used to give power supply and transmit the measured data to the serial server. (**b**) Ten minutes data compared with the vibratory capacitor meter in the OLED manufacturing process. The vibratory capacitor meter was installed next to our meter in the same gap distance to the glasses.

The meter was also installed into an OLED manufacturing line, to monitor the static voltage on the glass along with a traditional vibrating capacitor sensor, as shown in Figure 6a. Except for the meter, the test system also included an electrically grounded holder, a serial server for converting the RS485 protocol into a TCP/IP protocol, a computer, etc. The computer collected data from dozens of meters, and transmitted the data to the manufacturing management system for electrostatic safety management.

The MEMS-based meter, for measuring the OLED static charge, was installed next to a commercial vibrating capacitor-type meter for comparison. The minimum readout of the vibrating capacitor type was 10 V. After preparing the meters, cite calibration was also conducted, because the assembly structure was different from the lab calibration. We placed a metal plate on the roller, and applied a known voltage to the plate. In the case where the voltage was attenuated by the roller, an insulate Teflon plate was put between the metal plate and the roller. The magnification between the applied voltage and meter result was calculated as the geometrical coefficient. As shown in Figure 6b, the two meters’ measuring results agreed well, while the MEMS sensor curve was more elegant because of the better resolution. The actual test results show that the MEMS-based electric field sensor prototype has the potential to be used in the static charge monitoring in the OLED manufacturing processes. Nevertheless, both of the two kinds of sensors have their advantages and disadvantages. The vibrating capacitor’s electrodes are exposed to the air, which is robust in ionized air, but fragile from mechanical collision or low air damping. In contrast, the MEMS type’s electrodes are well protected by the package, the resolution is enhanced by the package, and the robustness is better in mechanical collision/low air damping, but worse in ionized air. Furthermore, with various features of MEMS technology, our proposed electric field microsensor will also be promising in terms of cost and mass production.

## 4. Discussion

In this paper, a new package structure for the electric field microsensor is proposed and applied into an electrostatic voltage meter. Being significantly different from the existent microsensor chip structure designs, our design focuses on the optimization of the structure of the package, and realizes a distinct improvement in the electric field resolution. The resolution is verified both by the charged plate test and the real test. Table 2 makes a comparison between the electric field resolution parameter of our work and several previous works.

The dynamic range of the proposed electric field microsensor obeys the Nyquist’s theorem, which indicates that the maximum detecting frequency of the microsensor is half of the resonant frequency. However, it takes a large number of calculations in the microprocessor to detect such a high-frequency electric field. Therefore, the actual detectable dynamic range is less than the theoretical value. Several approaches might be considered to improve this. First, use a better microprocessor that is stronger at math calculations. Second, increase the resonant frequency in the design of the micro structure, such as the dimension of the electrodes. Third, demodulate the sensor output with the double-frequency output, while the dynamic range will also be the double value.

Several drawbacks exist in this design, and still need further consideration. First, since the sensitivity is magnified, the measuring range is narrowed. Therefore, if a larger measurement range is required, the meter should be placed farther away from the object, meanwhile the sensitivity is lower then. Second, the package cap structure is also sensitive to the accumulated electrostatic charge, which might be transferred to the installation process. Although, in this paper, we neglect the accumulated charge and consider the package cap as an electroneutral conductor, it is not easy to keep neutral in the actual test. It takes hours, or even days, for the accumulated charge to dissipate.

The new proposed package structure is also available in other circumstances. For example, in the dusty atmosphere, traditional vibrating capacitor meters or the field mills are prone to be destroyed by the particles, and the hermetic sealed package is able to protect the sense mechanical structures. Furthermore, the package method can also be used for improving the resolution of other electric field sensors, such as vibrating capacitors, optical-type electric field sensors, and other types of MEMS-based electric field microsensors.

The temperature fluctuation is a factor for the drift of the sensor. When the temperature changes, the Young’s module of the silicon-based materials, the stress on the interface of the microsensor and the package shell, air damping, and humidity, will change, leading to the drift of resonant frequency, resonant amplitude, and the sensitivity. In this paper, the sensor prototype was settled in the clean room, where the control of the temperature was relatively stable, and the thermal drift was inconspicuous. For other applications, in order to improve the thermal stability, the closed-loop feedback circuit and microsensor need to be studied in the future.

## 5. Conclusions

Electrostatic voltage measurement is a key factor in the ESD control area. Rather than designing the sensor’s micro structure, here, we bring out a new package structure with a non-flat package cap. By inducing more charge under the electric field, and concentrating the charge to a narrow space, the electric field inside the package cavity is enhanced significantly. Both theoretical analysis and finite element simulation have proved this concept. Furthermore, based on the electrostatic microsensor, a high-resolution contactless voltage meter is also proposed. After a calibration, lab test, and real environment test, it was proven that our prototype had a resolution that was better than 3 V in a 2 cm distance, and it performs better than the traditional vibrating capacitor in the OLED production line. With various advantages of the MEMS technology, the proposed electrostatic voltage meter prototype is promised to be widely used in the future.

## Figures and Tables

**Table 1:** Parameters in the simulation model.

Part Number	Part Name	Radius (mm)	Height (mm)	Material	Relative Permittivity
①	Package shell	4	1.9	Ceramics	9
②	MEMS chip	2.5	0.5	Silicon	/
③	Air	3.3	1.55	Air	1
④	Inner electrode	0.8~3.2	0.1~0.9	Covar	/
⑤	Package cap	4	0.1	Covar	/

**Table 2:** Comparison of electric field resolution with previous works.

Electric Field Microsensors	Year	Resolution
Unpacked resonant chip in [24]	2003	630 V/m
Unpacked resonant chip [30]	2011	40 V/m
Packed sensor system [39]	2016	10 V/m
Unpackaged resonant chip [32]	2019	20.4 V/m/√Hz
This work by package design	2021	5 V/m
